# Whole-Genome Analysis of Quorum-Sensing Burkholderia sp. Strain A9

**DOI:** 10.1128/genomeA.00063-15

**Published:** 2015-03-05

**Authors:** Kok-Gan Chan, Jian Woon Chen, Kok Keng Tee, Chien-Yi Chang, Wai-Fong Yin, Xin-Yue Chan

**Affiliations:** a Division of Genetics and Molecular Biology, Institute of Biological Sciences, Faculty of Science, University of Malaya, Kuala Lumpur, Malaysia; b Centre of Excellence for Research in AIDS (CERiA), Department of Medicine, Faculty of Medicine, University of Malaya, Kuala Lumpur, Malaysia; c Interdisciplinary Computing and Complex BioSystems (ICOS) Research Group, School of Computing Science, Claremont Tower, Newcastle University, Newcastle upon Tyne, United Kingdom; d The Centre for Bacterial Cell Biology, Medical School, Newcastle University, Newcastle upon Tyne, United Kingdom

## Abstract

Burkholderia spp. rely on *N*-acyl homoserine lactone as quorum-sensing signal molecules which coordinate their phenotype at the population level. In this work, we present the whole genome of Burkholderia sp. strain A9, which enables the discovery of its *N*-acyl homoserine lactone synthase gene.

## GENOME ANNOUNCEMENT

Bacteria communicate through signaling molecules, a cell-cell communication known as quorum sensing (QS) (1, 2). *N*-Acyl homoserine lactone (AHL) is one of the common QS signaling molecules synthesized by *Proteobacteria* (3). Burkholderia spp. are pathogens often found in the lungs of cystic fibrosis patients, and uses AHL as the QS signaling molecule to communicate not only within the same species but also between the bacterial community residing in the human lung upon infection (4–6). The QS property of Burkholderia sp. strain A9 has been confirmed, but the gene responsible for its AHL production remains unknown (7). In view of this, we performed whole-genome sequencing of Burkholderia sp. strain A9 with the ultimate goal of searching for its AHL synthase gene.

Burkholderia sp. strain A9 was isolated from soil using a KGm medium and routinely maintained on a Luria-Bertani medium (7, 8). Bacterial genomic DNA was extracted with MasterPure DNA purification kit (Epicenter, USA) and subjected to next generation sequencing (NGS) sample preparation with a Nextera DNA library preparation kit (Illumina, USA) (9, 10). The sequencing library was quantified using Qubit 2.0 (Invitrogen, USA) and qualified with Bioanalyzer (Agilent, USA). The NGS was performed on MiSeq (Illumina, USA) (10). Sequencing raw reads were trimmed and assembled using CLC Genomic Workbench (v7.5) (11). Subsequently, the genome was annotated using NCBI prokaryotic annotation pipeline (v2.9) and BLAST against the NCBI nonredundant (NR) database (12, 13).

A total of 1.8 million reads were generated in this sequencing project. The draft genome of Burkholderia sp. strain A9 was assembled into 98 contigs with an *N*_50_ of 136,739 bp resulting in a genome size of 3.46 Mbps. The average coverage of this genome is 32-fold, and the G+C content is 65.62%. A total of 3,010 coding DNA sequences (CDS) were identified from this genome. The genome sequence of Burkholderia sp. strain A9 contains 3,128 genes, 89 pseudogenes, 5 rRNAs, and 23 tRNAs.

Our previous study confirmed that Burkholderia sp. strain A9 produces AHLs, namely, *N*-hexanoylhomoserine lactone and *N*-octanoylhomoserine lactone (7). In this genome study, an AHL synthase gene with a length of 609 bp was determined by analysis of the genome sequence. It is located at 194,270 to 194,878 bp of contig 16. The AHL-based QS of Burkholderia spp. regulates the expression of its extracellular proteins production, siderophores production, biofilm maturation, and swarming ability (14–16). Thus, with the availability of this whole-genome information, future work can focus on the importance of the QS of environmental Burkholderia sp. strain A9.

### Nucleotide sequence accession numbers.

The draft genome of Burkholderia sp. strain A9 was deposited into DDBJ/EMBL/GenBank under accession no. JSZN00000000. The version described in this paper is the first version, JSZN01000000.
